# Mortality, recovery, and community shifts of scleractinian corals in Puerto Rico one decade after the 2005 regional bleaching event

**DOI:** 10.7717/peerj.3611

**Published:** 2017-07-25

**Authors:** Jorge R. García-Sais, Stacey M. Williams, Ali Amirrezvani

**Affiliations:** 1 Department of Marine Science, Universidad de Puerto Rico, Recinto de Mayagüez, Mayagüez, Puerto Rico, Puerto Rico; 2 Institute for Socio-Ecological Research, Lajas, Puerto Rico, Puerto Rico; 3 Coastal Survey Solutions LLC, Lajas, Puerto Rico, Puerto Rico

**Keywords:** Shifts, Coral assemblages, *Orbicella annularis*, Caribbean, Coral reefs, Recovery, Mortality

## Abstract

This work analyzes the mortality, recovery, and shifts in the composition of scleractinian corals from Puerto Rico one decade after the 2005 regional coral bleaching event. Temporal and spatial patterns of coral community structure were examined using a stratified, non-random sampling approach based on five permanent transects per reef at 16 reef stations. A negative correlation between percent coral cover loss and light attenuation coefficient (Kd_490_) was observed, suggesting that light attenuation, as influenced by water turbidity and depth, played a major role in coral protection during the bleaching event (“sunblock effect”). Responses of coral assemblages varied after the bleaching event, including shifts of cover from massive corals (*Orbicella* spp.) to opportunistic (*Porites astreoides*) and branching corals (*Madracis auretenra*, *P. porites*) and/or turf algae; partial recovery of reef substrate cover by *O. annularis* complex; and no measurable changes in coral assemblages before and after the event.

## Introduction

Coral reef communities have been changing dramatically over the past four decades (Gardner et al., 2003; Hughes et al., 2003; Jackson et al., 2014). Natural and anthropogenic disturbances are detrimental to the ecological integrity of coral reefs and the abundance of reef-associated organisms. There have been major declines in large commercially important fish and shellfish, sea urchins and massive corals. Coral bleaching events are a recurrent phenomenon affecting coral reefs in the Caribbean (Goenaga, Vicente & Armstrong, 1989; Glynn, 1993; Winter et al., 1998; Williams & Williams, 2000; Wilkinson & Souter, 2008; García-Sais et al., 2008), with major events having occurred in 1998 (Winter et al., 1998) and more recently in 2005 (Hernández-Delgado et al., 2006; García-Sais et al., 2008; Weil, Croquer & Urreiztieta, 2009; Eakin et al., 2010). Long-term data quantifying the impacts of major bleaching events upon reef community structure are lacking in the Caribbean. Specifically, information on long-term responses, including recovery from large-scale mortality associated with regional bleaching events is of particular interest.

Coral bleaching is the whitening of corals due to the loss of their symbiotic algae. Reductions in salinity (Goreau, 1964; Bahr, Jokiel & Rodgers, 2015), decrease or increase in light (Jokiel, 1980; Lesser, 1996; Lesser et al., 1990; Gleason & Wellington, 1993; Brown, 1997; Torres et al., 2007; Torres, Armstrong & Weil, 2008) and an increase in sea surface temperature (Jokiel & Coles, 1977; Jokiel & Coles, 1977; Hoegh-Guldberg & Smith, 1989; Glynn, 1993; Ferrier-Pages et al., 2007) are factors that cause corals to bleach or turn pale. In areas exhibiting higher summer temperatures, higher variability in temperature, and/or a lower rate of seasonal warming, corals are less likely to bleach indicating that a higher rate of adaptation and/or acclimation may make them resistant to bleaching (Castillo & Helmuth, 2005; McClanahan et al., 2007; Mumby et al., 2011; Oliver & Palumbi, 2011; Chollett, Enríquez & Mumby, 2014). Several studies have proposed that there is a natural “refugia” with increasing water depth and water turbidity associated with inorganic and organic sources (West & Salm, 2003; Wilkinson & Souter, 2008; Bridge et al., 2013; Smith et al., 2014; Guest et al., 2016). In the Indian and Pacific Oceans, the impacts of coral bleaching are predicted to be less severe in areas of high organic turbidity (Van Woesik et al., 2012; Cacciapaglia & Van Woesik, 2015).

Turbidity can be caused by either inorganic (sedimentation) or organic sources (phytoplankton biomass), each having different environmental implications. Inorganic turbidity is caused by an influx of sediments from coastal development, river runoff, and/or the resuspension of materials in the benthos. The influx of phytoplankton does occur naturally, however, anthropogenic activities influence nutrient loading in coastal environments due to activities including, but not limited to, agriculture and/or sewage leaching. In the west coast of Puerto Rico, a positive correlation has been observed between turbidity and chlorophyll-a (Gilbes, López & Yoshioka, 1996), with western and near coastal coral reefs exhibiting higher levels of chlorophyll-a (Otero & Carbery, 2005). The north and west coasts receive most of the nutrient loading from the discharge of some of the largest rivers (Morelock, Grove & Hernandez, 1983), whereas rivers with smaller drainage basins are found along the south coast. There are no rivers in either of the offshore islands (Mona, Desecheo, Culebra, and Vieques), which are characterized by clear, oligotrophic waters (Morelock, Grove & Hernandez, 1983).

The most severe coral bleaching event reported for Puerto Rico and the US Virgin Islands occurred in late August 2005 (Hernández-Delgado et al., 2006; García-Sais et al., 2008; Weil, Croquer & Urreiztieta, 2009; Eakin et al., 2010), and coincided spatially and temporally with the passing of a mesoscale anticyclonic eddy across the northern Caribbean (García-Sais et al., 2008). Anticyclonic eddies are mesoscale systems that occur four to five times a year in the western tropical Atlantic and Caribbean basin (Alvera-Azcárate, Barth & Weisberg, 2009), introducing an important source of variability to the mean current (Gordon, 1967; Molinari et al., 1981; Carton & Chao, 1999; Andrade & Barton, 2000). These are convergent systems that tend to depress thermoclines and associated pycnoclines. Because of the downwelling motion, nutrients remain stratified deep in the water column below the pycnocline resulting in a highly oligotrophic surface mixed layer that allows for deep light penetration both in the visible and UV spectrums.

Coral bleaching was severe at many sites throughout the Caribbean during late 2005, affecting 50%–95% of coral colonies in some localities (Wilkinson & Souter, 2008). The effects on Puerto Rican coral reefs were variable between geographic locations, depths, and coral species (García-Sais et al., 2008). More than 90% of scleractinian corals in the northeastern coral reefs of Puerto Rico and the USVI displayed signs of thermal stress, becoming pale or bleached (Hernández-Delgado et al., 2006; Miller et al., 2009). *Orbicella annularis* complex was among the most impacted of all coral that bleached in 2005 (García-Sais et al., 2008; Hernández-Pacheco, Hernández-Delgado & Sabat, 2011), with more than 90% of the populations recorded as bleached in Puerto Rico and the US Virgin Islands (Ballantine et al., 2008; Miller et al., 2009).

Reports of coral recovery after massive bleaching events in the Caribbean are scarce (Idjadi et al., 2006; Manfrino et al., 2013; Muller, Rogers & Van Woesik, 2014). Idjadi et al. (2006) described the phase shift reversal exhibited by Dairy Bull Reef, Jamaica in which live coral cover doubled within a nine-year period (1995–2004) up to a maximum cover of 54% largely due to growth of *Acropora cervicornis*. In Little Cayman Island, full recovery of corals was seen seven years after the bleaching event in protected areas (Manfrino et al., 2013). The recovery of corals at Little Cayman Island was attributed to the low anthropogenic disturbance at the reef site. The increase of anthropogenic stressors, such as coastal development, eutrophication and sedimentation, and loss of key reef herbivores have been associated with the lack of coral recovery at many Caribbean coral reefs (Carilli et al., 2009; Wilkinson, 2010; Roff & Mumby, 2012).

In this paper we describe the response variability, including the mortality, recovery, and shifts in composition of scleractinian corals on reefs included in the Puerto Rico Coral Reef Monitoring Program (PRCRMP-DNER/NOAA), one decade after the 2005 regional coral bleaching event and analyze the relationships between organic turbidity and coral loss and recovery.

## Materials and Methods

### Study sites

**Figure 1:**
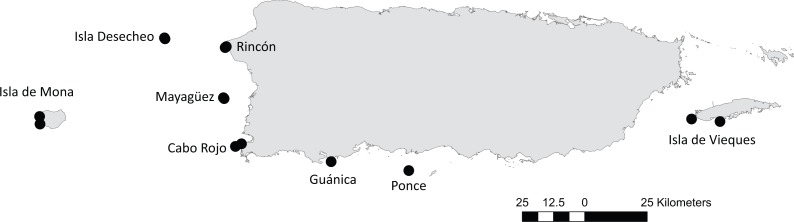
Location of 16 reef stations from eight areas in Puerto Rico: Isla Desecheo, Isla de Mona, Rincón, Mayagüez, Cabo Rojo, Guánica, Ponce, and Isla de Vieques.

A total of 16 reef stations were included in this time series analysis: Isla Desecheo (hereafter Desecheo), Isla de Mona (hereafter Mona), Rincón, Mayagüez, Cabo Rojo, Guánica, Ponce, and Isla de Vieques (hereafter Vieques). Depth-stratified samplings were performed at Desecheo (15, 20, 30 m), Mona (10, 20 m), Mayagüez (10, 20, 30 m), Rincón (10, 20 m), Cabo Rojo (5, 10 m), and Vieques (10, 20 m). Reef stations in Desecheo, Mona, Mayagüez, and Ponce represent offshore/shelf-edge sites, whereas reefs at Rincón, Guánica, Cabo Rojo and Vieques are representative of coastal sites (Fig. 1). Reef station locations and site names are listed in Table 1.

**Table 1:** Geographic coordinates, depths and monitoring years for Natural Reserve coral reef sites in Puerto Rico. Abbreviations for reef stations are listed.

Natural reserve	Sites names	Latitude	Longitude	Location	Depth (m)	Years
Isla de Vieques	Esperanza	18.298	−65.560	Offshore	10	2001, 2004, 2012, 2016
	Canjilones	18.189	−65.698		20	2001, 2004, 2012, 2016
Ponce	Derrumbadero	17.904	−66.609	Shelf-edge	20	2005–12, 2015, 2017
Guánica	Cayo Coral	17.936	−66.888	Inshore	10	2005–12, 2015, 2017
Cabo Rojo	El Palo	18.001	−67.211	Inshore	5	2000, 2012, 2015
	Resuellos	17.991	−67.233		10	2000, 2012, 2015, 2017
Mayagüez	Tourmaline	18.166	−67.276	Inshore	10	2004–12, 2015, 2017
	Tourmaline	18.165	−67.275		20	2004–12, 2015, 2017
	Tourmaline	18.163	−67.274		30	2004–12, 2015, 2017
Rincón	Tres Palmas	18.347	−67.270	Inshore	10	2004–12, 2015, 2017
	Tres Palmas	18.350	−67.266		20	2004–12, 2015, 2017
Isla Desecheo	Puerto Botes	18.382	−67.488	Offshore	15	2004–12, 2015
	Puerto Botes	18.382	−67.489		20	2004–12, 2015
	Puerto Canoas	18.378	−67.487		30	2004–12, 2015
Isla de Mona	Las Carmelitas	18.099	−67.938	Offshore	10	2000, 2008–10
	Playa Mujeres	18.072	−67.937		20	2000, 2008–10

### Benthos

At each reef, a set of five permanent 10 m long replicate transects were non-randomly established to represent reef areas of optimal coral growth within similar depths (+/−2 m) and reef physiographic zones. Transects were permanently marked with metal rods drilled to the reef substrate at both ends and nails were placed along the length of each transect. The installation of transects varied between sites from 1999 to 2004, and some of the sites were not monitored annually. Sessile-benthic reef communities were characterized by the continuous intercept chain-link method (as modified from Porter, 1972), following the CARICOMP (1984) protocol. This method provides information on the percent linear cover by sessile-benthic biota and other substrate categories along transects. The number of chain links per substrate category and species was added and then divided by the total distance (total number of chain links) to calculate the cumulative percent linear cover by each substrate category.

### Water quality measurements

Satellite-derived water quality products were used to examine their potential relationship to live coral degradation and/or recuperation. Diffuse attenuation coefficient for downwelling irradiance at 490 nm (Kd_490_) (Mueller, 2000; Lee et al., 2005) was used as an index of turbidity and was derived from Sea-Viewing Wide Field-of-View Sensor (SeaWiFS) (O’Reilly et al., 1998; O’Reilly et al., 2000; Maritorena & O’Reilly, 2000), and Moderate Resolution Imaging Spectroradiometer (MODIS) data. The SeaWiFS and MODIS Aqua satellite Kd_490_ and chlorophyll-a annual data were used from 2000 to 2013 to determine trends in water quality.

The study area was 4°× 4°, from 16°to 20°N latitude and 68°to 64°W longitude. Level 2 satellite products (only available at daily temporal resolution) were selected over Level 3 products (available at daily, monthly, and yearly temporal resolutions) since higher spatial resolutions are required for the study of water quality in coastal areas. The spatial resolution of SeaWiFS Level 3 data is 9 km and 4 km while for MODIS Aqua is 9 km. SeaWiFS and MODIS Level 2 data. Selection of coral reef sites’ pixel locations was made first by collecting the latitudes and longitudes (lat/lon) as well as depths of each respective site. Representative pixels were then selected to avoid the influence of land pixels and pixels influenced by ocean bottom reflectance signal. The lat/lon of the pixels used were selected along a line tangent to the shelf edge and starting at each coral reef site. The lat/lon used was based on where the ocean bottom was not visible while being as close as possible to each coral reef site.

SeaDAS v6.4 software was used to perform data visualization (Baith et al., 2001) and to accurately project and ortho-rectifiy the daily Kd_490_ data files into the same Conic Lambert’s map projection output. Only data files with minimal or no pixel distortions were selected for the study. Daily Kd_490_ data files picked were re-visualized, using MATLAB version 7.12.0 (The MathWorks Inc., Natick, MA, USA). This included the transformation of daily data into monthly and annual averages. The reef sites’ Kd_490_ values were extracted from their respective lat/lon pixel values using the average annual data files.

### Statistical analyses

#### Coral cover

Two-way Repeated Measures Analysis of Variance (ANOVA) procedures were run to compare percent reef substrate cover by live scleractinian corals and specifically of percent cover by *Orbicella annularis* complex (hereafter *Orbicella annularis*) between depths and years at each reef. Year and depth were assigned as the within subject factor for the analyses. There were some locations where depth was not a factor (Guánica and Ponce). In those instances, a One-way Repeated Measures ANOVA analysis was used to test variations of percent total coral cover and *Orbicella* spp. cover between years. The assumption of normality was analyzed before running any tests. Coral cover was arcsine transformed before analyses. Bonferroni post-hoc procedures were performed to examine yearly variations of percent coral cover at each site. Repeated measures ANOVA tests were run in SPSS software (version 17.0).

#### Coral assemblages

Two-way distance Permutational Multivariate Analyses of Variance (PERMANOVA) tests (Anderson, 2001) were performed to examine changes in coral assemblages between depths and years at each reef. At reef sites where depth was not a factor, a one-way PERMANOVA analysis was used to test variations in coral assemblages between years. Each PERMANOVA procedure was based on Bray-Curtis similarity measures. Coral species that contributed to more than 10% of the total cover were used for this analysis. *A posteriori* pairwise comparison tests were performed in PERMANOVA to further assess variations within the factors. SIMPER tests were run to identify the contribution of coral species to the overall differences between years. Multidimensional scaling (MDS) plots were produced to examine differences in coral assemblages between years. PERMANOVA, SIMPER, and MDS procedures were performed using PRIMER-e and PERMANOVA add-on software (Anderson, Gorley & Carlke, 2008).

#### Relationships between changes of coral cover and diffuse attenuation coefficient (*Kd*_490_)

Given the nonlinearity nature of the coral data, we used generalized additive models (GAM) to analyze the relationship between changes in coral cover at the upper reef depth surveyed (≤15 m depth) and satellite-derived Kd_490_ light attenuation coefficients from the immediate vicinity of the reef stations. Coral cover was the response variable, while Kd_490_ was treated as the fixed factor. To select the optimal structure of the random component, we ran two models, the first with no random term, and the second using sites as the random component. The optimal model was chosen based on the lowest Akaike’s Information Criteria (AIC) value by using the MuMln package. A smoothness selection was fitted by maximum likelihood through the Laplace approximation. All analyses were performed in R v.3.1.1 using package mgcv v1.8-0. Wood (2006) for GAM.

## Results

### Pre-bleaching event conditions

**Figure 2:**
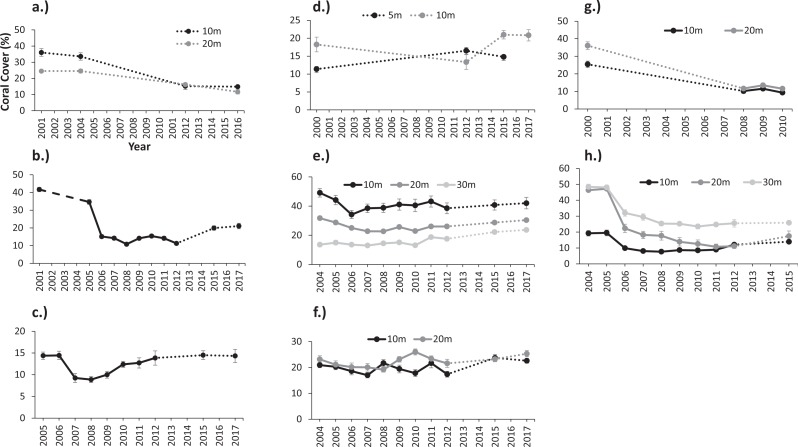
Variations of mean coral cover (%) between monitoring surveys at: (A) Isla de Vieques, (B) Ponce, (C) Guánica, (D) Cabo Rojo, (E) Mayagüez, (F) Rincón, (G) Isla de Mona, and (H) Isla Desecheo in Puerto Rico. The dashed lines signify years in which no data were collected.

Oceanic and shelf-edge reefs had the highest percentages of live scleractinian coral cover (hereafter live coral) during the baseline surveys performed before the onset of a regional coral bleaching event in August 2005. Mean live coral cover ranged from 32% to 52% at reef stations from Desecheo (20 m and 30 m), Mona (20 m), Ponce, and Mayagüez (10 m and 20 m). Coral cover at coastal reefs in Guánica, Cabo Rojo, and Rincón ranged from 10 to 20%, while at the coastal reefs of Vieques ranged from 26 to 36% (Fig. 2). Live coral cover increased with depth at Desecheo, but declined with depth at Mayagüez (Figs. 2E and 2G).

*Orbicella annularis* (complex) exhibited the highest percent cover by live corals at all reef sites except at Rincón 10 m, where *Montastraea cavernosa* was the dominant species. The relative contribution of *O. annularis* to the total coral cover at reefs ranged between 31.4% (Mayagüez 10 m) and 79.8 % (Vieques 10 m) and largely contributed to the similarity of coral assemblages within reef stations.

### Post-bleaching event dynamics

**Figure 3:**
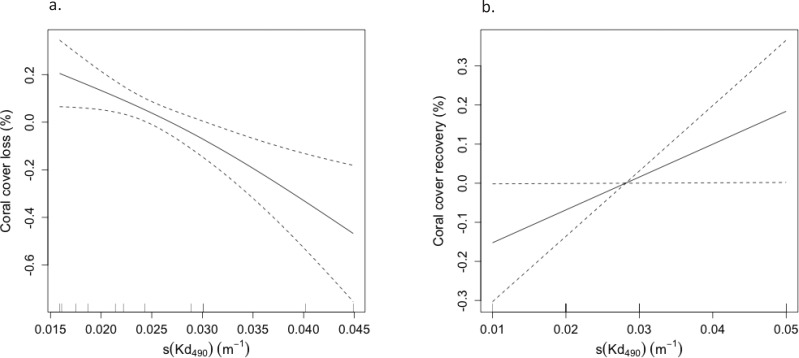
(A) Negative correlation between coral cover loss (%) and attenuation coefficient (Kd_490_) during 2006 and 2008 and (B) positive correlation between coral cover recovery (%) and light attenuation coefficient (Kd_490_) from 2009 and 2012 at reef stations in Guánica, Mayagüez (10 m), Ponce, and Isla Desecheo (15 m).

An inverse relationship was observed between coral mortality and light attenuation coefficient (Kd_490_) (Table 2, Fig. 3A). Offshore and shelf-edge waters were characterized by clear, oligotrophic water, with yearly means of Kd_490_ ranging from 0.018 m^−1^ at Desecheo to 0.024 m^−1^ at Ponce during 2005. Statistically significant declines of live coral cover ranging between 36% and 52% were measured during the 2006 annual monitoring survey from oceanic reefs in Desecheo, across all depths surveyed (Fig. 2, Table 3). Significant reductions in coral cover were also measured at Ponce (68%), Vieques (33.3% at 10 m and 58.6% at 20 m), Guánica (38.2%), and Mayaguez (29% at 20 m). Progressive declines of live coral cover were measured at many of the reef stations until 2008. Even though our post-bleaching surveys at Mona, Vieques, and Cabo Rojo reef sites occurred several years after the 2005 coral bleaching event, measurements of coral mortality at these sites by Hernández-Delgado et al. (2006) and García-Sais et al. (2008) provide additional support to the contention that coral decline at these reefs was indeed associated to the 2005 bleaching event. These studies concluded that the amount of bleaching varied among these reefs. Nearly 75% of the corals were partially or fully bleached at Cabo Rojo, while close to 60% of the corals were bleached at Mona Island. According to Hernández-Delgado et al. (2006), corals of the east coast, including those from Vieques were some of the most severely impacted, with estimated bleaching frequencies reaching as high as 95%.

Minor declines of live coral cover were observed at Mayagüez 10 m (21.0%), but differences were not statistically significant (Table 3). Changes of live coral cover between pre- and post-bleaching surveys were not detected from Mayagüez 30 m, reef stations at Rincón (10 m and 20 m) and Cabo Rojo (5 m and 10 m). In 2005, inshore sites were characterized by low light attenuation, with yearly mean values of Kd_490_ ranging from 0.036 m^−1^ at Mayagüez to 0.056 m^−1^ at Guánica.

**Table 2:** Results of the generalized additive model (GAM) to assess the relationship between coral cover loss (%) and attenuation coefficient (Kd_490_) during 2006 and 2008 at reef stations in Guánica, Mayagüez (10 m), Ponce, and Isla Desecheo (15 m). Sites were selected as random factors, while Kd_490_ values were fixed.

Coral loss	Estimate	Std. error	*z*-value	*p*-value	*R*-sq. (adj)
Intercept					0.90
	3.77	0.12	30.23	<0.0001	

	Estimate	Std. error	Chi.sq	*p*-value	
s(Kd_490_)	1.35	1.57	9.41	0.01	
s(site, random)	2.60	3.00	28.14	<0.0001	
AIC	81.55				

**Table 3:** Results of two-way and one-way repeated measures ANOVA procedures testing the variations of coral cover between depths and year at the different natural reserve reef sites in Puerto Rico.

Site	Factor	Type III SS	*df*	MS	*F*	*p* value
Vieques	Depth	2.76E−06	1	2.76E−06	0	0.99
	Year	1.03	3	0.34	7.98	0.003
	Depth*Year	0.15	3	0.15	3.81	0.123
Ponce	Year	0.88	9	0.10	7.99	<0.0001
Guánica	Year	0.32	9	0.04	1.89	0.08
Cabo Rojo	Depth	0.08	1	0.08	23.75	0.01
	Year	0.04	2	0.02	2.88	0.11
	Depth*Year	0.02	2	0.01	1.35	0.31
Mayagüez	Depth	4.11	2	2.05	15.76	0.002
	Year	0.35	10	0.03	5.47	<0.0001
	Depth*Year	0.33	20	0.02	1.69	0.053
Rincón	Depth	0.07	1	0.07	1.32	0.31
	Year	0.15	10	0.01	1.35	0.24
	Depth*Year	0.08	10	0.01	1.73	0.11
Desecheo	Depth	1.51	2	0.76	26.33	<0.0001
	Year	1.02	9	0.11	17.62	<0.0001
	Depth*Year	0.62	18	0.04	4.75	<0.0001
Mona	Depth	0.02	1	0.02	1.40	0.3
	Year	0.31	3	0.10	55.66	<0.0001
	Depth*Year	0.02	3	0.01	0.89	0.48

Depth related variations of live coral losses were measured from reefs surveyed at Desecheo, Mayagüez and Vieques. At Desecheo, live coral declined 60% and 62% at 15 m and 20 m, respectively, while the decline at 30 m was 43% (Fig. 2G). At Mayagüez, live coral cover declined 21% and 28% at 10 m and 20 m, respectively, but no measurable difference was observed at 30 m. Live coral cover from reef stations in Vieques declined 58.6% at 10 m and 33.3% at 20 m.

The 2005 coral bleaching event had a severe impact on the mortality of *O. annularis*. Statistically significant reductions of substrate cover by *O. annularis* were observed at all depths from oceanic island reefs; Desecheo and Mona, the Ponce shelf-edge reef, Mayagüez 10 m, and the coastal reefs of Vieques (Table 4). The relatively high contribution of *O. annularis* to the total scleractinian coral composition of oceanic and shelf-edge reefs strongly influenced the overall decline of total live coral at these reefs. Conversely, differences of live cover by *O. annularis* were not statistically significant at coastal reefs of Cabo Rojo, Rincón, and the deeper sections of the Mayagüez (30 m) shelf-edge reef.

### Coral recovery

Recovery trends of coral cover were noted from several reef stations after 2008. By 2015, full recovery of live coral loss were recorded from Guánica and Mayagüez 20 m, and coral cover increased by 95.2% at Mayagüez 10 m. Positive increases of live coral cover have been measured at the Mayaguez 30 m and at the Rincón reefs (158% and 120%). Increases of coral cover were slower at shelf-edge and offshore reefs relative to coastal sites. A positive correlation between coral cover recovery (2008–2013) and Kd_490_ was observed (Table 5, Fig. 3B).

**Table 4:** Two-way and one-way repeated measures ANOVA procedures testing the variations of reef substrate cover by *Orbicella annularis* (complex) between depths and year at the different natural reserve sites surveyed in Puerto Rico.

Site	Factor	Type III SS	*df*	MS	*F*	*p* value
Vieques	Depth	0.50	1	0.50	3.88	0.12
	Year	1.64	3	0.55	6.94	0.006
	Depth*Year	0.13	3	0.04	1.47	0.27
Ponce	Year	2.56	10	0.26	11.50	<0.0001
Guánica	Year	0.43	9	0.03	1.66	0.13
Cabo Rojo	Depth	0.13	1	0.13	1.94	0.24
	Year	0.25	2	0.12	2.85	0.12
	Depth*Year	0.32	2	0.16	2.48	0.15
Mayagüez	Depth	5.20	2	2.60	15.02	0.002
	Year	3.60	10	0.36	31.88	<0.0001
	Depth*Year	1.08	20	0.05	3.89	<0.0001
Rincón	Depth	12.01	1	12.01	6.82	0.059
	Year	0.30	10	0.03	1.45	0.19
	Depth*Year	0.40	10	0.04	1.14	0.36
Desecheo	Depth	13.09	2	6.54	31.26	<0.0001
	Year	9.10	9	1.01	20.04	<0.0001
	Depth*Year	1.41	18	0.08	1.99	0.02
Mona	Depth	0.15	1	0.15	0.55	0.5
	Year	4.00	3	1.33	8.96	0
	Depth*Year	0.81	3	0.27	3.31	0.06

**Table 5:** Results of the generalized additive model (GAM) to assess the relationship between coral cover recovery (%) and attenuation coefficient (Kd_490_) from 2009 to 2012 at reef stations in Guánica, Mayagüez (10 m), Ponce, and Isla Desecheo (15 m). Sites were selected as random factors, while Kd_490_ values were fixed.

Coral recovery	Estimate	Std. error	*z*-value	*p*-value	*R*-sq. (adj)
Intercept	4.08	0.10	42.04	<0.0001	0.83

	Estimate	Std. error	Chi.sq	*p*-value	
s(Kd_490_)	1.00	1.00	4.09	0.04	
s(site, random)	2.55	3.00	38.42	<0.0001	
AIC	112.40				

The magnitude of coral recovery also varied with depth. At Desecheo, coral cover increase was greater at 15 m (71.7%), but moderate increases were also measured at 20 m (37.0%) and at 30 m (52.7%). By 2017, coral cover increased by 60.8% at Ponce. Coral cover has not increased at reef sites in Vieques and Cabo Rojo 10 m. The lack of recent surveys from Cabo Rojo 5 m and Mona reef sites limit our assessment of potential recovery at these reef sites.

### Changes in coral composition

**Figure 4:**
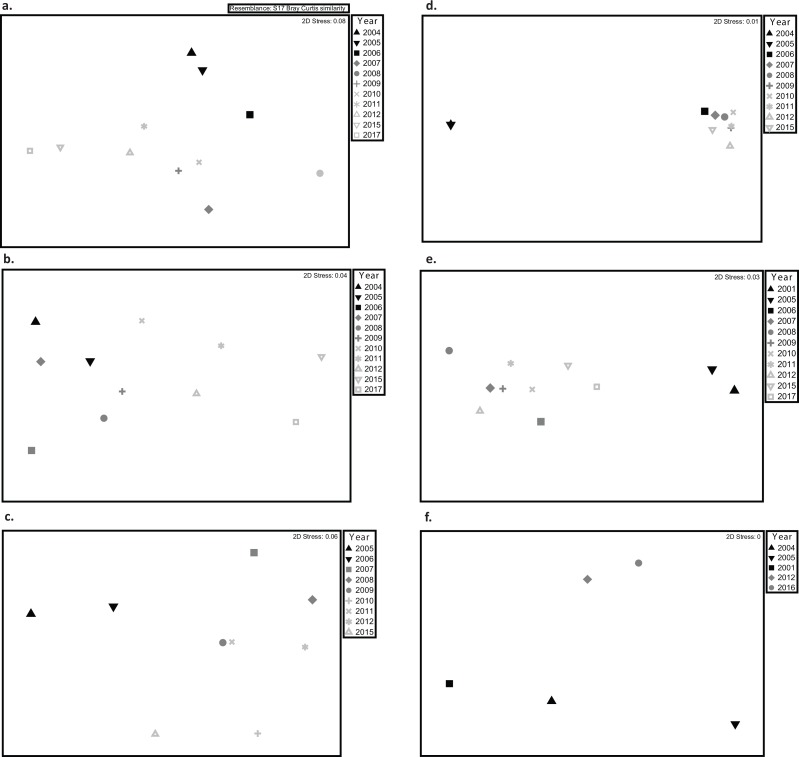
Multidimensional scaling (MDS) plots showing variations in coral assemblages between monitoring surveys at (A) Mayagüez, (B) Rincón, (C) Guánica, (D) Isla Desecheo, (E) Ponce, and (F) Isla de Vieques. Given the lack of interaction between year and depth, depths were pooled for visualization purposes. Due to the low sampling years, data from Isla de Mona and Cabo Rojo were excluded from the MDS. Graphs display distance of centroids with year as the grouping factor.

**Figure 5:**
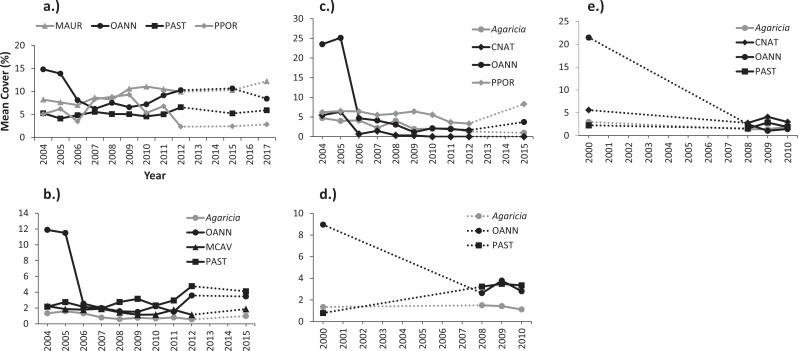
Variations of mean percent cover by coral species during monitoring surveys at: (A) Mayagüez 10 m, (B) Isla Desecheo 15 m and (C) 20 m, and (D) Isla de Mona 10 m and (E) 20 m. Coral codes are OANN, *Orbicella annularis* (complex); PAST, *Porites astreoides*; PPOR, *P. Porites*; MAUR, *Madracis auretenra*; and CNAT, *Colpophyllia natans*.

Marked variations of coral assemblages were observed at reefs severely affected by the 2005 coral bleaching event, including those at Desecheo, Mona, Vieques, and Ponce (Table 6, Fig. 4). These reefs were all affected by the sharp decline of cover by *O. annularis*. Variations were dependent on reef geographic location and depth. At Desecheo 15 m, reef substrate cover by *Porites astreoides* and *Agaricia* spp. was not negatively impacted by the bleaching event and consistently increased through time (Fig. 5B). Despite the partial, yet continued recovery trend of *O. annularis* during recent years (2013 to present), *P. astreoides* now stands as the main coral species in terms of substrate cover at Desecheo 15 m (Fig. 5B). A similar pattern was observed at Desecheo 20 m, but the main phase shift of coral dominance was towards *P. porites,* replacing not only *O. annularis* but also *Colpophyllia natans,* which ranked second in cover during the pre-bleaching surveys, yet suffered drastic mortality during 2006 (Fig. 5C). At Desecheo 30 m, the change in composition was mostly related to the demise of *C. natans,* and the increase in relative abundance of *P. astreoides* and *Agaricia* spp., whereas *O. annularis* remained the dominant species after the bleaching event.

**Table 6:** Permutational Multivariate Analysis of Variance (PERMANOVA) procedures testing the variations of coral composition between years and depths.

Site	Factor	SS	*df*	MS	Pseudo-*F*	*p* value
Vieques	Depth	1262.10	1	1262.20	0.92	0.45
	Year	16731.00	4	4182.80	3.06	0.001
	Depth*Year	8300.60	3	2766.90	2.02	0.022
Ponce	Year	21210.00	10	2121.00	2.96	0.001
Guánica	Year	10521.00	9	1169.00	0.66	0.958
Cabo Rojo	Depth	2246.60	1	2246.60	1.67	0.142
	Year	51541.20	3	1713.70	1.28	0.248
	Depth*Year	3679.90	2	1839.90	1.37	0.207
Mayagüez	Depth	1.25E+05	2	62609.00	72.96	0.001
	Year	9769.00	10	976.90	1.14	0.253
	Depth*Year	12748.00	20	637.39	0.74	0.972
Rincón	Depth	54164.00	1	54264.00	39.56	0.001
	Year	10036.00	10	1003.60	0.73	0.929
	Depth*Year	5238.60	10	523.86	0.38	1
Desecheo	Depth	87185.00	2	43593.00	29.50	0.001
	Year	35250.00	9	3916.70	2.65	0.001
	Depth*Year	25523.00	18	1418.00	0.96	0.612
Mona	Depth	6247.10	1	6247.10	3.42	0.007
	Year	14760.00	3	4920.00	2.69	0.004
	Depth*Year	5582.10	3	1860.70	1.10	0.449

Shifts of coral assemblages were prominent at Mona reef stations, where *O. annularis* cover decreased to levels below *P*. *astreoides*, a species that was resilient to the bleaching event (Figs. 5D and 5E). There were statistically significant declines in live coral cover by *O. annularis* (Table 6) at Ponce and Vieques reefs, but the loss of coral cover was largely replaced by turf algae at these reef stations. Changes in coral dominance were also evident at Mayagüez 10 m (Fig. 5A). The statistically significant decline of cover by *O. annularis* was replaced by branching species, such as *Madracis auretenra* and *P. porites* during a five-year period (2007–2011). Since then, a consistent recovery trend of *O. annularis* has been measured, reaching present levels of cover similar to those of *M. auretenra* and higher than those of *P. porites*, which has declined in mean cover at Mayagüez 10 m since 2011 (Fig. 5A).

Coastal reefs of Cabo Rojo, Rincón, Guánica and deeper reef stations of Mayagüez (20 m and 30 m) did not exhibit any significant differences in coral assemblages associated with the 2005 bleaching event. Moderate declines of substrate cover by *O. annularis* were observed at Guánica and Mayagüez 20 after the bleaching event but since have recovered to pre-bleaching conditions.

## Discussion

Puerto Rican coral reefs have changed significantly during the last decade, both regarding the total live coral cover and the relative composition of scleractinian coral assemblages. Localized impacts upon coral reefs associated with anthropogenic activities have been reported (Hughes et al., 2003; Fabricius, 2005; Warne, Webb & Larsen, 2005), but the main driver of change on the Puerto Rican coral reef community has been linked to the 2005 regional coral bleaching event (García-Sais et al., 2008; Bruckner & Hill, 2009). Given the spatial context and long-term data collection of this study, we determined that depth, distance from shore, and reef location relative to riverine discharges were relevant factors influencing the resilience variability of corals in the different reefs to the bleaching event.

Elevated sea surface temperature (SST) and time of exposure to the SST anomaly were proposed as the main drivers of the 2005 coral bleaching event (Brown, 1997; Hoegh-Guldberg, 1999; Eakin et al., 2010). Fluctuating temperature regimes may allow for greater thermal acclimation at the nearshore environments (Chollett, Enríquez & Mumby, 2014). However, coral reefs surveyed in this study were distributed within the water column surface mixed layer and therefore exposed to similar water temperatures within the 10–30 m depth range (Sprintall & Tomczak, 1992; Armstrong, Lopez & Gilbes, 2000; Corredor & Morell, 2001). A distinct and consistent pattern of reduced coral mortality with increasing depth was observed at reefs surveyed from various depths, including those at Desecheo, Mona, Vieques and Mayagüez, suggesting that light may have played a determinant role as a precursor of coral bleaching and subsequent mortality, perhaps in synergy with elevated SST during the 2005 bleaching event.

**Figure 6:**
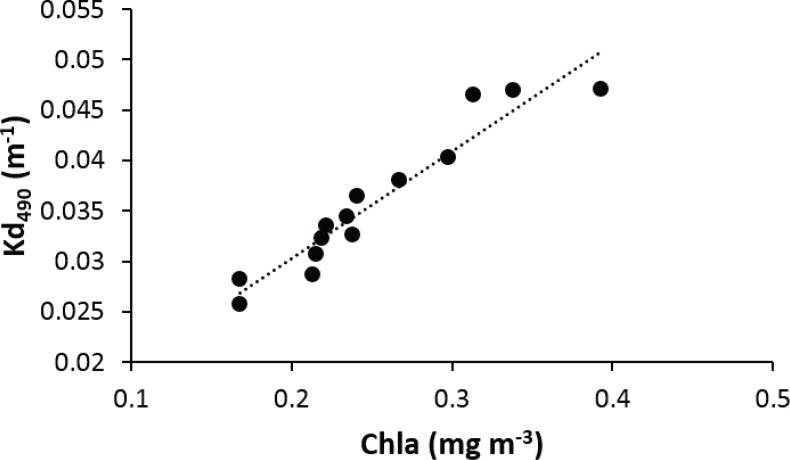
Linear regression showing a positive relationship between the average chlorophyll-a and attenuation coefficient (Kd_490_) in the vicinity of reefs surveyed in Puerto Rico from 2000 to 2013.

Light penetration decreases exponentially with depth, but is also affected by water turbidity from both inorganic and organic sources. Inorganic suspended sediments can increase turbidity in specific areas affected by riverine inputs. In this study, the main driver of water turbidity variations along the insular shelf and nearby offshore waters around Puerto Rico was chlorophyll-a concentrations, an indicator of phytoplankton biomass (Fig. 6). Due to the oligotrophic conditions of oceanic waters surrounding Puerto Rico, nutrient loadings associated with riverine inputs and other coastal estuarine processes convey for pronounced and distinct neritic-oceanic gradients of water turbidity (Schmuker, 2000 Otero & Carbery, 2005; García-Sais et al., 2008).

Contrary to the generalized paradigm that coastal reefs subjected to higher levels of environmental stressors may be more vulnerable to degradation, these were more resilient to the 2005 regional coral bleaching event than their offshore (shelf-edge) and oceanic counterparts. Oceanic and shelf-edge reefs and reefs located upstream from riverine inputs in this study were the most severely affected by the bleaching phenomena and showed slower recovery of live coral cover compared to coastal and/or downstream reefs. For example, Tourmaline Reef is located at the shelf edge off Mayagüez Bay and thereby influenced by estuarine conditions and higher water turbidity than the oceanic reefs of Desecheo and Mona on the west coast of Puerto Rico. The marked contrast of live coral loss at the 30 m depths from reef stations at Desecheo 30 m (43%) versus Mayagüez 30 m (0%), suggests that distance from shore and the inherent increase of water turbidity towards the coast may have also played a role in coral protection. Likewise, the negligible coral loss observed from the coastal reefs of the Cabo Rojo and Rincón shelf, influenced as well by estuarine conditions, is in sharp contrast to the significant mortalities observed at similar depths from reefs in Vieques, located far from the influence of major rivers. These patterns were supported by the negative relationship measured between coral cover loss and light attenuation (“sunblock effect”).

Of particular concern due to potentially irreversible consequences for Puerto Rican coral reef communities is the fact that *Orbicella annularis* (complex), the main reef building coral of Puerto Rican reefs and main contributor to overall coral cover, was severely affected by the coral-bleaching event. Drastic declines in *Orbicella* spp. after the 2005 bleaching event were also reported at Navassa Island (Miller, Piniak & Williams, 2011). The large-scale degradation of *O. annularis* has implications for structural habitat loss due to bioerosive forces, both in terms of the overall topographic relief and habitat complexity and a loss in overall carbonate production (Edmunds & Elahi, 2007; Alvarez-Filip et al., 2011; Alvarez-Filip et al., 2013; Perry et al., 2013). The susceptibility of *O. annularis* to the synergistic effect of elevated water temperature and high levels of ultra-violet radiation is well documented (Goreau & Macfarlane, 1990; Szmant & Grassman, 1990; Castillo & Helmuth, 2005; Manzello et al., 2007; Hernández-Pacheco, Hernández-Delgado & Sabat, 2011; Bayraktarov et al., 2013). Increased protection of live coral cover by *O. annularis* was observed with increasing depth at oceanic and shelf-edge reef sites, as well as at coastal reef stations with higher water turbidity.

The variation of resilience by different coral species to the bleaching event led to changes in coral assemblages at reef stations. Essentially, four major patterns of coral community structure have been noted since 2005 by this monitoring program; (1) a marked decline of cover by massive corals (*Orbicella* spp.) replaced by a proportional increase in cover by turf algae; (2) a shift of coral assemblages from massive to opportunistic and branching species (*Porites astreoides*, *Madracis auretenra*, *P. porites*), (3) a partial recovery by *O. annularis*; and (4) no measurable change in coral assemblages before and after the bleaching.

Community shifts to opportunistic corals have been previously reported (Green, Edmunds & Carpenter, 2008) and this is due to their inherent life history traits such as short longevity (Soong, 1991), brooding larvae, and relative high fecundity (Chornesky & Peters, 1987; McGuire, 1998). Brooding corals have the capability to survive disturbances (Green, Edmunds & Carpenter, 2008). The shift to weedier coral species can have serious ecological impacts on coral reef function, resulting in the loss of coral calcification and structural complexity (Alvarez-Filip et al., 2013).

Due to the lingering effects associated with coral disease infections, most reef sites reached their minimum coral cover by 2008. However, one decade after the bleaching event, coral recovery trends have been measured from impacted reef sites. There have been reports of coral recovery after bleaching events from many parts of the world (Baker, Glynn & Riegl, 2008; Gilmour et al., 2013; Graham et al., 2013; Manfrino et al., 2013), however this is the second study to report large scale (multiple reefs) coral recovery in the Caribbean after a major disturbance. In Puerto Rico, reef decline has been attributed to the increase of sediment discharge and nutrient inputs along the south and west coasts of Puerto Rico (Morelock, Grove & Hernandez, 1983; Warne, Webb & Larsen, 2005). However, we suggest that increased water turbidity by organic sources may have protected corals from bleaching, and the higher plankton biomass affecting water turbidity nearshore may have functioned as an available food source for corals to promote faster recovery due to potentially higher growth rates (Wooldridge, 2014). This premise is reinforced by other studies in the Indian and Pacific Oceans (Van Woesik et al., 2012; Cacciapaglia & Van Woesik, 2015), which observed turbid nearshore environments as climate change refuges with turbidity mitigating high-temperature bleaching. In addition, there have been reports of healthy coral reefs (high coral cover) in the Caribbean in areas of high anthropogenic inputs (García-Sais et al., 2014; López-Victoria, Rodríguez-Moreno & Zapata, 2015).

## Conclusion

The bleaching event in 2005 was one of the worst disturbance on record affecting coral reef communities in the Caribbean, with particularly pronounced impacts on *Orbicella annularis*, the main reef building coral species. Variable resilience of coral species to bleaching were noted, as well as a spatial pattern of increased coral recovery associated with increasing depth, nearshore, and downstream location relative to riverine discharges. Likewise, a variable pattern of coral recovery was observed, with faster recovery associated with nearshore reefs. These data suggest that light attenuation, as influenced by organic turbidity and depth played an important role in the protection of corals during the bleaching event (sunblock effect). Also, the higher plankton biomass nearshore may be influencing coral recovery. By 2015, there was an increase in coral cover at the majority of the reef sites surveyed driven by increases of reef substrate cover by *O. annularis* and/or overgrowth of dead massive coral sections by branching opportunistic species.

##  Supplemental Information

10.7717/peerj.3611/supp-1 Supplemental Information 1 Index 1 Scleractinian coral cover at the different sites, depths and years.

10.7717/peerj.3611/supp-2 Supplemental Information 2 Index 2 *Orbicella annularis* complex coral cover at the different sites, depths and years.

10.7717/peerj.3611/supp-3 Supplemental Information 3 Coral species cover The cover of the different coral species between the reef sites in Puerto Rico. Coral species listed contributed to at least 10% of the total cover.

## Additional Information and Declarations

### Data Availability

The following information was supplied regarding data availability: The raw data has been supplied as a Supplementary File.
